# Working hard to belong: a qualitative study exploring students from black, Asian and minority ethnic backgrounds experiences of pre-registration physiotherapy education

**DOI:** 10.1186/s12909-019-1821-6

**Published:** 2019-10-16

**Authors:** John A. Hammond, Annabel Williams, Saskia Walker, Meriel Norris

**Affiliations:** 1 Faculty of Health, Social Care and Education, Kingston University and St George’s University of London, Cranmer Terrace, London, SW17 0RE UK; 2 Department of Clinical Sciences, Brunel University London, London, UB8 3PH UK

**Keywords:** Physiotherapy, Student, Black Asian and minority ethnic, Qualitative, Race

## Abstract

**Background:**

Previous research has demonstrated that attainment inequalities exist for students from Black Asian and Minority Ethnic (BAME) groups in pre-registration physiotherapy education. While previous research has explored students from BAME backgrounds experience of university, the context of physiotherapy is unique and is under researched. Therefore the purpose of this study was to explore BAME student experiences during their physiotherapy training.

**Methods:**

Using a phenomenological approach pre-registration BSc and MSc students from BAME backgrounds from two universities who had completed both academic and clinical modules were invited to participate. Focus groups followed a topic guide developed from the literature and were facilitated by physiotherapy educators from outside the host institution. They were digitally recorded, transcribed verbatim and analysed thematically. Analytical triangulation was adopted throughout the research process as a mechanism to enhance rigour.

**Results:**

Seventeen students participated from a range of self-identified BAME backgrounds that were also representative of age, gender and course. Themes derived from the data included: feeling an outsider in reflections of belonging, behaviours by others that marginalise BAME and personal strategies to integrate in physiotherapy despite the lack of power and influence. Collectively these themes demonstrate a range of challenges which students from BAME backgrounds face within both an academic and practice learning environment.

**Conclusions:**

While this may not be surprising based on other disciplines, this study demonstrates that studying physiotherapy as a student from BAME background requires persistence to overcome a series of many implicit challenges. Understanding the experiences of students from BAME backgrounds presents unique opportunities to educate the profession and co-create opportunities for a more diverse profession with practitioners and educators as role models. There is a need for greater training for educators to listen to these students’ voices and their stories, and understand where institutional structures and practices could be modified to enable BAME student inclusion in physiotherapy education and practice.

## Background

In recent decades, there has been a significant focus on widening participation to higher education in the UK and worldwide, leading to greater numbers of people studying a higher degree. Yet there has been growing evidence in higher education to suggest that there are differential levels of attainment for those students from different ethnic backgrounds even when other demographic variables are adjusted for [1, 2]. This has also been demonstrated in healthcare education [3–7] and specifically physiotherapy in the USA [8] and more recently in the UK [9].

While these studies demonstrate an attainment gap they do not explain why this occurs. Some studies have tried to explore this through various methods such as surveys, interviews and video analysis of specific interactions. Broadly these studies identify that Black, Asian and Minority Ethnic (BAME) individuals are more likely to feel more isolated or not academic enough [2, 10]. Findings also demonstrate BAME students are less likely to participate in extracurricular activities due to cultural or family reasons [11]. Steele [12] suggests BAME underperformance can been explained by the theory of ‘stereotype threat’ where an individual from a negatively stereotyped group may feel threatened or anxious in the context of ethnic majority and thus may affect performance. Other research confirms BAME attainment differences in clinical examinations and claim that no overt discrimination was evident from either video analysis of the exam [13] or comparison to anonymously marked work [14].

However these findings emphasise a deficit model that locates the issue with the individual [15, 16]. A deficit focus may lead only to remedial strategies targeting the individual (such as supplementary courses [17]) and ignore the broad social factors involved inadvertently excusing discriminatory practices and structures [18, 19]. More recently there has been greater acknowledgement that institutional structures and implicit bias may contribute to attainment inequalities [20]. As a result many institutions have developed a multi-factorial change programme to address attainment gaps [2, 21]. However this broader perspective also has its critiques, suggesting that a change programme may still perpetuate inequality and misrecognition of BAME experience [22].

Physiotherapy presents a unique perspective due to its particular history in the UK and worldwide context. The physiotherapy profession has traditionally been perceived as white and middle class [23] however figures from 2017 to 18 demonstrate 19% of all UK physiotherapy programme student enrolment identify from a BAME background [24]. While this is a greater proportion of the population than most recent census data (14%) from 2011 [25], it is still lower than the proportion of BAME students applying 21.53% [24]. Recent evidence suggests that applicants from some BAME backgrounds have little knowledge of physiotherapy [26] and indicate it has lower status in their communities [26, 27]. The nature of the physiotherapy course and the culture of the profession presents some unique issues for some cultural and faith groups, such as expectations of touch and physical handling of clients and varying levels of undress for both learning and practice. These issues have been identified pre-entry [26] and there is some evidence of the challenges at the intersection of gender and culture during the programme [28]. One study by Haskins [29] demonstrated covert bias when physiotherapists assessed a video recording of physiotherapy students of different ethnic backgrounds reciting identical scripts – rating Black, Hispanic and Asian students lower than white students. Inequality may also extend in seeking meaningful employment, where physiotherapy graduates emphasise the burden of assimilation into the predominantly white culture of the healthcare service [30]. Apart from these studies, there is limited exploration of how students from BAME backgrounds experience or negotiate these factors throughout their physiotherapy education, making it difficult to know what and how to address it. A focus on the idiosyncrasies of a professional course such as physiotherapy has the potential to illuminate the complex intersection of factors that influence experience and avoid an assumed ‘one size fits all’ approach. This research aims to explore this phenomenon from the experience and perspective of current physiotherapy students from BAME backgrounds in the South East United Kingdom (UK).

## Methods

This study was conducted in parallel with research exploring experiences of students with disabilities [31]. Informed by phenomenological approaches [32] this qualitative study focused on the BAME students’ experiences throughout all aspects of the course.

Ethnic group categories used by the Higher Education Statistics Agency (HESA) [33] were adopted to ensure consistency with national demographics. In this paper the term Black, Asian and Minority Ethnic (BAME) is used to describe those groups who describe themselves other than white. Categorisation like this is not ideal, as many heterogeneous groups are included together and we acknowledge this may be a limitation from the outset.

### Sample

The study was conducted at two Higher Education Institute (HEI)‘s in the South East of England for practical reasons. The total combined pre-registration physiotherapy student population was approximately 500. We were interested in the experiences of physiotherapy students who self-identified as being from a BAME background and based on the proportion of 19% previously identified, a pool of 95 potential participants was estimated. Students from both BSc and MSc pre-registration courses at the two HEI’s were invited to participate in the study via a cohort wide email. Those students who volunteered and had completed both academic modules and clinical placements represent a convenience sample [34].

### Data collection methods

To facilitate collective and shared responses prompted through interaction, focus groups were selected [35]. A focus group at the penultimate (MSc year 1 and BSc year 2) and final (MSc year 2 and BSc year 3) level were conducted at each HEI. They followed a topic guide which included; discussion about what participants consider their own ethnic identity, their experiences in university and placement and how ethnic identit (ies) may have impacted, and using our previous research findings as a framework [9] we asked the participants to offer explanations of the attainment differences and how it might be addressed. All focus groups were facilitated by experienced qualitative researchers who were also physiotherapy academic staff (MN with AW as co-facilitator, JH with SW as co-facilitator). We acknowledge that none of the researchers identified from a BAME background, however are representative of the academic staff demographics in each HEI and more broadly the profession. To ensure that the participants felt able to share details about all aspects of the university including staff, the interviews were conducted by researchers from outside the participants’ institution. The focus groups were digitally-recorded and to aid collective member checking key points were noted on a flipchart during the focus groups. This process helped to avoid researcher assumptions by clarifying meanings and enabling participants to prioritise areas.

### Analytical strategy

Digital recordings were transcribed verbatim and anonymised. Using a thematic analysis framework [36] all researchers read and familiarised themselves with the transcripts and two researchers (JH, AW) independently assigned initial codes, categories and super-ordinate themes. Following critical discussions with all co-researchers, the lead researcher (JH) reviewed and refined the themes in an iterative process to enhance the depth and transparency of analysis [37].

## Results

The sample included 17 students (10 F, 7 M, 13 BSc, 4 MSc) with a range of ethnic backgrounds including Black, Asian and Mixed backgrounds. The focus groups lasted approximately 90 min. See Table 1 for participant details.

**Table 1:** Focus group participant details

Participant number	Focus Group^a^	Gender	Course^b^	Year of study	Ethnic background^c^
1	J3	M	MSc	2 (Final)	Mixed – White and Black Caribbean
2	J3	F	MSc	2 (Final)	Black or Black British - Caribbean
3	J3	F	BSc	3 (Final)	Black or Black British - Caribbean
4	J3	M	BSc	3 (Final)	Black or Black British - African
5	J3	M	BSc	3 (Final)	Other Black background
6	J3	F	BSc	3 (Final)	Black or Black British - African
7	J3	F	BSc	3 (Final)	Black or Black British - African
8	K3	M	MSc	2 (Final)	Black or Black British - Caribbean
9	K3	M	BSc	3 (Final)	Black or Black British - African
10	K3	F	MSc	2 (Final)	Black or Black British - African
11	J2	F	BSc	2 (penultimate)	Mixed - White and Black African
12	J2	F	BSc	2 (penultimate)	Black or Black British - African
13	J2	M	BSc	2 (penultimate)	Black or Black British - Caribbean
14	K2	F	BSc	2 (Penultimate)	Black or Black British - African
15	K2	F	BSc	2 (Penultimate)	Asian or Asian British - Indian
16	K2	F	BSc	2 (Penultimate)	Chinese
17	K2	M	BSc	2 (Penultimate)	Other Asian Background

Legend: ^a^Focus group – institutional identifiers J and K, number represents final [3] or penultimate [2] year group ^b^BSc = BSc (Hons) Physiotherapy degree, MSc = pre-registration MSc Physiotherapy degree ^c^ethnic background self-identified by participant with reference to HESA ethnicity categories

Three major themes developed from the analysis of the data of participants experiences. The first theme **‘you do feel like an outsider’** focuses on the participants’ sense of belonging in physiotherapy education. **‘Everyone wants to shy away and tip-toe around it’** is the second theme and explores the overt and subtle practices of others (particularly tutors, peers and practice educators) that marginalise BAME students. Finally the theme **‘we don’t have much power and influence’** illustrates the personal strategies that are adopted by individuals from BAME backgrounds to establish a sense of belonging in physiotherapy education. In tandem, these themes drawn from the participant experiences also highlight where institutional structures and practices could be modified to enable BAME student inclusion and these are presented in the discussion.

### Theme 1 - ‘You do feel like and outsider’: feeling ‘other’ in reflections of belonging in physiotherapy

This theme explores the perceptions of participants from BAME backgrounds in relation to (what they identify as) the default physiotherapy student identity, being ‘white, middle class, mature and female’. They suggest that this is something they perceived is embedded in a history of physiotherapy and only becomes apparent at the commencement of the course. The participants indicate the acute realisation that there is less BAME representation in physiotherapy in comparison to other aspects of their lives and prior educational experiences. As a consequence their feelings of being an outsider in physiotherapy education are amplified, as this participant identifies:

“at the beginning, when I started … .there was a lot of, like, Asian people on other courses –like, medicine and biomed, and then when I was, interacting with them, I felt I was on the wrong side (laughter), cos I was in physio, which is the main, like, white people, I was maybe I’m on the wrong course, I should be on the other side” K2, p27

The BAME participants in this study often report that they perceive their professional behaviour is being judged in relation to this default physiotherapy identity. This has significant personal and social implications for them and they articulate this through a process of belonging in physiotherapy education.

“cos you do already feel sort of like an outsider … … … You’re always conscious of that … .. like how are they perceiving me? How am I being?, you know?” J2, p6

“I mean, when I talk to all the … . lecturers and staff here, and they talk about their background, where they’re from, you pick up little things here and there that they’re from these nice villages or nice towns and cities where they had a very nice house, erm, front and back garden and, you know, they never had issues with heating or water or, you know, anything like that that you seem to think do you know what?, they’re, they’re, they might not understand where I’m coming from” K2,p20

This last statement demonstrates how other aspects of identity such as class and socio-economic status intersect with ethnic background, in addition to the obvious power differential between staff and students. Participants interpret the academic institution, and their peers, place a higher value on (what they perceive to be middle class and white) modes of communication that further amplifies their feelings of difference. In addition, the participants feel disadvantaged in that this is not their mode of communication, which leads them to feel isolated, disadvantaged, and stigmatised as these participants highlight

“I think I was speaking quite normal like I don’t even know what normal is, but I think I was speaking like alright and they [class peers] were just laughing at everything I was saying … … .everything I was saying they was like (laughs), everyone started laughing. I was thinking, “I didn’t say a joke here like,” but you know what I just took it” J3 p15

“so now, like, we subconsciously just think, like, if we ever like speak in front of the class and we pronounce something wrong that they’ll really laugh about it afterwards” K2, p54.

The identification of the default physiotherapy identity by participants goes beyond obvious demographic variables, and includes other characteristics and values. The participants describe an ideal ‘extrovert’ physiotherapy type that they attribute with characteristics such as: being outgoing, flamboyant, socialising and going to the pub, being sporty or proactive and motivated. The respondents feel vulnerable when attempting to adopt the ‘extrovert’ physiotherapy identity as they may be positioned negatively based on cultural stereotypes

“ … ..there’s certain, like, things you can’t say and certain, like, ways that you have to sort of, like, hold yourself because as a black person it’s easier to be misconstrued as being either loud or aggressive or rude” J2, p7

By contrast, another participant feels their quiet approach, which they align with their ethnic background, will be judged negatively (isolating myself) in comparison to the default ‘forthcoming’ approach.

“other students who were not of colour but they were more, maybe more forthcoming with their information, very active, umm, in the team in terms of oh, you know, asking what they’re gonna do after work and stuff like that, like I’m very, I’m a, sort of a remote worker, like if I’ve got work to do I’ll just crack on and get on with it, and I think they didn’t really like that and they interpreted that as me sort of isolating myself” K3, p7–8

Overall the participants tend to cluster a series of characteristics that they perceive to be highly valued and create an imagined identity that will be privileged in physiotherapy education. This results in them feeling a deficit, and positioned as an outsider, which is intensified in physiotherapy compared to other aspects of their lives. Consequently this impacts on the development of their professional identity as an authentic physiotherapist. .

### Theme 2 ‘Everyone shies away and tiptoes around it’ – other peoples’ responses and behaviours that marginalise BAME

This theme focuses on the participants’ perceptions of the responses of the university staff, practice educators and peers in a variety of contexts relevant to their study as a physiotherapy student. The participants report practices that make them feel included in the physiotherapy community, however these are often isolated and rare. More commonly the participants reflect on practices of ethnic majority staff, peers, practice colleagues that demonstrate ignorance (lack of understanding), denial / avoidance or ‘microaggressions’ that suggest both advertent and inadvertent racism.

Firstly participants describe a number of responses of staff/peers that they interpret as indicative of a lack of understanding of ethnic/cultural difference. Examples are expressed of where course content highlights ethnic differences and ethnic majority teaching staff discuss it awkwardly or in a way that amplifies the difference.

“when they [lecturers] try to make it, try not to be racist and you can just see it, you can see it, like they’re trying not to do it so hard that it just turns into it, they try so hard, like they try and make it so normal, like, umm, there was one time we were sitting in tutorial class and [lecturer] was like, “Yeah, I’m saying the word ‘black’ you guys shouldn’t be worried I’m saying the word ‘black’ … . black is normal … ” There was no need for that explanation and [the lecturer] was looking at me, as she was saying she was looking at me as sort of like she was trying to support me, like she’s trying to … … … I’m thinking, “It’s fine like just say it like.” And I was like, stuff like that when you’re trying not to do it, it just turns a lot worse it just becomes a lot more awkward, and everyone, everyone all notices it, everyone’s like, “Woah, that was a bit different.” J3, p12

Rather than respect and value BAME diversity, the students talk of how these examples only tend to ‘put a massive magnifying glass over us’ J3,p11. And as previously expressed the participants identify that this emphasises how staff and peers do not understand their context from the often privileged perspective.

When issues related to BAME difference occur, the participants mostly describe responses by staff that are interpreted as dismissive or avoiding. For instance the following example recalls where a student raises concerns about racism.

“There was a module that we had to do. It was, uh, what was it called? Clinical Preparations, and I had to do, like … .,what are my threats?, … and I mentioned it … .. being someone who is black I’m scared that there might be racism and I had a white female person in a white coat with me and she was like, “well, that doesn’t happen”. I was like, well, how would you know? How could you possibly know that that doesn’t happen?” J2, p6

And similarly in practice, this black male student recalls an educator response that appears well meaning but avoids confronting the issue.

“so there’s certain umm, patients that won’t even like look at me. And my educator was open she was like, “Oh, you might not want to go into this with me, cos she’s a bit funny.” And I think, “What do you mean?” She’s like, “She’s a bit funny with men.” I was like, “Hmm, okay cool.” And then I realised and there was 3 physios I saw before and they were all men and I was like, “Okay are you sure it’s … ” “Okay, yeah, she’s a bit funny with like black men,” J3, p18

There are some behaviours observed in others that some students refer to as ‘microaggressions’. One participant describes these microaggressions as actions of ethnic majority people based on assumptions of race/ethnicity “instead of yourself as a person” J3, p13. Examples include direct physical behaviours such as hair touching

“They’ll say something, they’ll touch my hair. They’ll do that and then I’ll just be like why do you have to do that? Just like, and just, basically just put in a situation where you’re isolated by drawing attention to the fact that you’re different and it just happens all the time and it’s not done because they think that they are … They would never believe that they have these preconceptions … .”J2,p19

The findings also highlight examples of where staff and peers prioritise interactions, including verbal and non-verbal communication, with other people more like themselves (e.g. white).

“so, the woman [tutor] was like, Caucasian, and we were, it was just us three [students who were from BAME background] and we were like, everyone else was Caucasian, she was making eye contact with everyone else except us and we felt really left out (laughter) but I don’t know why she wasn’t making eye contact with us?, but, like, is it the race or … ..”K2, p12

These highlight where participants feel isolated in physiotherapy education. There are other situations where microaggressions are perceived at having a significant impact on their academic success. For instance students talk about perceptions of lower expectations of Black, Asian and Minority Ethnic students.

“I do feel like sometimes when I’ve got feedback for like an exam, umm, and it’s not been like a terrible grade but I just wanted to do better and one of the lecturers was like surprised that I like, received this grade and I wasn’t happy about it. And then for someone else [another student] like she told me that she didn’t get, like, she basically just passed and it was like, “Oh.” The lecturer was like, “Oh, this isn’t like you, like I’m really surprised that you haven’t, you know, done better. Like what’s the problem?” And I just thought like, “What other reason would there be?” This was right at the beginning as well, like why would he just assume that I didn’t want to do better? J3,p14

This can lead to perceptions of difference in marking practices and this student talks about differences between her experience as a black woman on placement compared to a white peer:

“We literally had exactly the same responsibilities because it was very similar work. It was just in two different wards … ..and we took on the same role, blah, blah, blah, and she came out with a grade that was 10% higher than mine … … … Yeah, and part of that was the fact that she got on so well with her clinical educator. Like literally, like, (*laughter*) they could be sisters at this point and they just walk along together and me and my clinical educator we look very different and so it’s just, it’s just interesting. I don’t know why, but maybe due to the fact we didn’t have that immediate connection where we have something to, you can’t, like, look in the mirror and then, like, reflect each other’s thing. Maybe that was a thing … … .but I think definitely part of that would be the fact that I already had a barrier or being black and it’s not something that I particularly like” J3,p20.

It is important to recognise that this example is based on the participant’s perception. It is impossible to determine what the clinical educator’s motives were, but for the student, that shared sense of belonging through ethnicity was not present. This example and the others illustrated in this theme indicate that from the participant’s perspective, BAME difference is either dismissed, avoided or illuminated through microagressions in many physiotherapy contexts.

### Theme 3 ‘We don’t have much power and influence’ - personal strategies by students from BAME backgrounds to integrate

This theme arose from the participant discussions and from a recognition that they feel disempowered in physiotherapy education. The participants in the study claim “we don’t have much power and influence” J2,p25 and they highlight an impression that they “don’t know how much can change” K2,p59 and from their position as students it is difficult to challenge because they are always under the pressure to meet the requirements of assessment. Nevertheless the findings indicate personal strategies to establish a sense of belonging in physiotherapy education and these are expressed in contrasting ways. Some strategies confront the perceived default physiotherapy identity by asserting their own ethnic identity. On the other hand, the findings identify strategies of self-regulation that either avoid challenge or conform uncomfortably to the perceived default physiotherapy identity.

To establish a sense of belonging students seek out others within their peer group that they feel they will have an affinity based on other aspects of identity.

“when it comes to studying now, you kind of look for the people who look like you to study – it’s really weird, I can’t explain it, I can’t put an exact reason why I do it but I just, it’s just maybe me, I just see it as: if I’m revising with this guy they might know the same amount that I do, we might be on the same equal footings, so let’s revise together because you won’t make me feel stupid” K3, p16

This example illustrates how creating an equal footing is not just establishing academic equivalence, but that it is also social and cultural. These attempts of belonging extend beyond the programme as one participant talked about seeking mentors or role models in practice and being surprised of the lack of black representation to choose from

“so I didn’t really see it [physiotherapy] as a race issue and even discussing it with family, friends, people that I’d come across, I think the only time that I sort of picked up that there weren’t a lot of female black women that were doing it when I was researching was when I was looking for mentors and people to actually talk to” K3,p5

The following represents one student’s attempts at asserting his own identity in the physiotherapy classroom to challenge the perceived assumptions made related to his ethnicity:

“I like being in a full tracksuit, coming in and then being able to answer every question, just maybe to plant the seed that just because I’m this way doesn’t mean that I’m stupid or anything. I don’t like stereotypes, I like to challenges stereotypes, so I always am conscious of that; I think umm, in the groups, you know, some people might come off as patronising sometimes, umm, the way I say things like, “Water” and I guess like all the time I can’t *“War-ter” or “but-ter”,* I say, “Water, butter,” it’s just how I speak. Whereas as soon as you say those type of buzzwords, someone else over there might be like, “Okay, this guy can’t even speak properly, I might need to take the reins here.” K3p19

This student also recognises that he needs to regulate his own behaviour and the findings suggest that students use a variety of strategies to modify and regulate their behaviour as this student indicates.

“It’s just you have to … … … ..Regulating your behaviour … … … Yeah, you’re constantly criticising what you’re doing. You’re constantly being like, okay, I can’t do that, I can’t say that or, okay, I have to be like this or I have to speak like this” J2p7

Others talk of strategies to avoid conflict including; staying silent, ignoring comments, staying calm, not getting upset and just taking it. This black female student talks about how she manages a racist comment in practice:

“I just take it as it’s just the way it is; so it’s kind of like you acknowledge it but you just move on, you don’t really give it too much energy, so, but I dunno if that’s, I dunno if that’s err, the right thing to do or the wrong thing to do, but at the end of the day that’s, yeah … ..Yeah, at the time just what else can you do? I mean especially on placement you’re just trying to get through placement to be honest, you’re not really trying to change the world there” K3,p4

Apart from being aware of how they might modify their verbal expression, the students also talk about the impact on how they feel they have to demonstrate skills in a particular ways. In one situation a student reflects on differences between himself, as a black man, within a white placement team and follows this by talking about how he modified his behaviour to manage:

“My reflections, my true reflections, I always write two, I write the one I’m gonna show, and I write one for myself, cos the one where I can be really honest and even write honest things, speak in a certain way, and umm, you know, it’s normally reflecting on just my experiences with the team, what I need to do next; if everyone focuses on what they need to do to pass the book, whereas I can focus on how I, how I can manage the team type thing” K2, p8

And another talks about how he modifies his behaviour with white patients

“And I think in terms of the way you act … … .I’ve had like similar things, elderly patients they don’t really want to umm, be, interact with a black person. And then sometimes you will try and change your approach so you’ll be extra … like we’re nice people cos we’re physios, we’ll be extra-extra nice to like an elderly white person trying not to seem aggressive or, but I mean you see other white physios will go in and tell their patients off, shouting at their patients, and it’s like if we ever did that sort of thing … … . we’d be seen as aggressive” J3,p19

These personal strategies of managing ethnic difference in physiotherapy education carry the significant emotional labour as another student articulates:

“but I don’t know about you but I think it’s emotional labour to have to constantly chat with people’s microaggressions just because they don’t know. So, like, there are certain things like sometimes somebody will come up and say something ignorant and I’ll challenge it. Don’t say that because of this, this and this. It’s offensive because of this, but once you’ve done that for 20 times a day for the past week, then it comes to, let’s say, the eighth day and you’re like, do you know what I’m gonna let this one slide and then it gets to a thing so, like … I was gonna say it’s about picking your battles.” J2p26

This theme draws on the personal burden that participants from BAME backgrounds experience in physiotherapy education and wider contexts, and how they adopt varying strategies which either assert their identity or assimilate by modifying behaviours.

## Discussion

The findings represent one of the first known studies to explore the experience of physiotherapy students from BAME backgrounds during their studies. We acknowledge that this is a small sample but as participants are from two institutions, this may help to avoid conclusions that the findings are specific to one context. We have taken the qualitative data as a whole, we recognise the sample is heterogeneous and interactions between other factors such as social class, gender or previous educational backgrounds may also influence our findings. Nevertheless the challenges perceived by the participants in this study highlight the occurrence of inequality in physiotherapy education. Although this research did not apply Critical Race Methodology [38], the principles of critical race theory [39] have been used as a lens to discuss the findings in the context of the structural and cultural aspects of physiotherapy education.

The narratives of the students from BAME backgrounds in this study might be what Solorzano and Yosso [38] refer to as ‘counter-stories’ of those people whose experiences are not often told. Therefore these voices of students from BAME backgrounds offer a way of exposing, challenging the stories of the majority and privilege in physiotherapy as the participants had desired. The participants, in this study, discuss an acute awareness of a position as an ‘outsider’ to what they perceive as white dominance within physiotherapy in comparison to other healthcare profession courses. They articulate attempts to belong in physiotherapy, a profession which they perceive places value on specific characteristics such as being extrovert and proactive, something that can be in conflict with their racial/cultural backgrounds. The participants then feel at a disadvantage and they have to work harder to demonstrate these characteristics. This has previously been highlighted by other researchers [14, 38] where demonstration of ideal characteristics is synonymised with right/good, and simultaneously implies what is not ideal/right (in this case introvert, quiet). This leads to a constant sense of needing to work to fit in and the sense of ‘not fully belonging’ is particularly significant for BAME students in physiotherapy. These feelings reflect other literature in higher education where students from BAME backgrounds have reported marginalisation and isolation [10, 16, 40] or not feeling academic enough [2]. One of the key findings of our research is that the data indicates that the context of training to be a physiotherapist, and the profession itself, intensifies the feelings of marginalisation and isolation for BAME students. This intensity of feeling had not been anticipated prior to engagement with the profession and was significantly higher in this context than the other areas of life experienced by the participants.

A second theme arising from the study was in relation to the responses of others through the participant’s physiotherapy education journey. The participants described experiences where issues of race/ethnicity were either ignored or denied and these reflect the experiences of social marginalisation that Beagan [40] describes in her study of medical student minorities in Canada. Madriaga [22] also argues that ‘meritocracy’, ‘value-neutral’ and ‘colour blind’ discourses pervade liberal majority higher education institutions, and that these uphold white privilege by being perceived as ‘natural’. Whether intentional or not, such discourses disregard those who are racialised as BAME (ibid), and in the case of the present study it appears that the majority (white) staff, peers and patients are indifferent to the realities of the marginalised students from BAME cultural backgrounds. There were examples of blatant racial/ethnic discrimination by patients in the practice context, which illustrate some of the challenges experienced by students and educational professionals, as they develop therapeutic interactions. However more often there were common, minor, every day acts based on stereotyped assumptions of ethnicity or race that convey disrespect, disregard or contempt that the participants and others refer to as ‘microaggressions’ [41, 42]. Examples of microaggressions have been identified in medicine [40] and the wider student community [41]. The examples of hair touching, avoiding eye contact, asking ‘where are you ‘really’ from?’ and assuming BAME students may have lower academic ability in the present study are not dissimilar to this literature. Therefore this draws attention to the issue of cultural marginalisation of BAME students in the classroom and practice context of physiotherapy education. The source of microaggressions are also widespread, including fellow peers [11], patients [43] and also those in positions of power such as teachers and clinicians [14, 40, 44]. The cumulative burden of microaggressions entrenches power relations and results in institutional racism that continues to marginalise BAME students in physiotherapy.

Despite the challenges identified in the study, the participants reveal their resourcefulness in attempts to integrate in physiotherapy. To avoid the consequences of being judged based on stereotyped assumptions; the students describe how they make efforts to be proactive, assertive or ‘extra nice’ which are similar to strategies identified in other work in physiotherapy and nursing [30] and in medicine and biomedical sciences [11]. But largely these strategies indicate attempts to modify behaviour to be ‘more like white’ and include other actions such as dressing like other students, modifying accents and avoiding confrontations. Beagan [40] argues that for self-preservation it is easier to not make a fuss and allow incidents to pass, as students can be perceived as over reactive or too sensitive by the privileged majority who are ignorant to the issues faced. Solorzano & Yosso [38] highlight that students of colour have no option but to assimilate to white middle class culture to succeed in university and life. Whilst this study recognises the need for all trainees to assimilate into the context of physiotherapy education, it also reveals some of the unique tensions experienced by BAME students. Consequently, the findings suggest these tensions make the assimilation more difficult for BAME students, highlighting the inequality of experience within the system.

The students in the study were keen to put forward recommendations for addressing the issues they faced and these have relevance in the context of the institutions that participated in the study, but others may draw on these examples. The participants stressed the need to educate staff and fellow peers of the contexts where privilege and disadvantage coexist. Stevenson [18] also argues that academic development is necessary so that staff learn to recognise white privilege and feel empowered to deal with issues of race and racism. The students were happy for their narratives to be used as part of these developments and we are identifying physiotherapy specific staff development. We are also currently exploring ways to co-create teaching, learning and assessment approaches [18, 19] with students from BAME backgrounds. Smith [2] has recently argued the importance of greater representation and role models and we are also developing approaches to establish BAME mentors.

These practices may go some way to give students from BAME backgrounds a greater sense of belonging. However others argue that gains will be minimal if the white dominate narrative remains unchallenged [39] and strategies are remedial and shaped on an assumption of deficit [19, 43], such as assuming the mentorship programmes alone will solve the issue. There is also need to recognise the system of higher education recreates privilege and entrenches power in systematic ways [22] and thus physiotherapy education is embedded within this system. To enable change, physiotherapy educators may need to challenge their own and institutional practices even if this means a willingness to let go of power. Beagan [40] claims from her work with medical education that by not questioning and challenging practices, educators are complicit in enabling marginalisation and inequalities for BAME and other identities. Despite these calls to action, it is not possible to identify whole scale inclusive approaches for ‘all’, because although well meaning, Madriaga [22] suggests such approaches promote a liberal ethic that ‘everybody is the same’ which simultaneously ignores experiences of students of colour.

## Conclusions

The findings in this study help to identify for the first time the unmoderated student perspective where structures, curriculum, teaching and learning strategies operate to simultaneously advantage ethnic majority students and disadvantage BAME students in physiotherapy education. Some of these are not dissimilar to other research, but this study highlights some microaggressions that are prevalent in physiotherapy education and how qualities such as extroversion and proactiveness may be privileged in physiotherapy limiting some cultural and ethnic approaches. It also highlights how students from BAME backgrounds demonstrate resourcefulness in attempts to belong in physiotherapy. Yet there is still much work to do to empower BAME students to speak up and advocate that their voices are heard. Students studying towards a physiotherapy degree are required to demonstrate adept academic and clinical skills, alongside all qualified physiotherapists, who are also bound in both behaviour and knowledge by regulatory frameworks (such as the Health and Care Professions Council Standards of Proficiency [45] in the UK). The complexity of the student experiences in this study are also impacted by the power imbalances in both the clinical and academic workspace, and in their ability to successfully negotiate and manage complex issues relating to BAME in a ‘professional’ way.

We hope the themes prompt colleagues to recognise similarities in their own environments and draw their own conclusions and actions. From these findings it is essential that academic practitioners and senior managers continue to interrogate education differentials related to ethnicity. Overt incidents of racism should also be dealt with via appropriate workplace policies and educators should publicly demonstrate intolerance of racism. Critically, BAME students should be empowered to tackle these overt forms of racism as well as the more subtle and oppressive microaggressions. A starting step could be to commit to, and invest in, activities that seek to co-create teaching, learning and assessment approaches with BAME students. By doing this, BAME students would be empowered to disrupt and change any overt or covert racist pedagogic practices. In addition, the lack of visible role models from BAME backgrounds in the profession also poses challenges. We suggest that focus and investment should be directed towards the identification and support of BAME role models and mentors within physiotherapy education, and across the profession. A greater diversity in role models and mentors may assist in making the profession attractive and accessible to potential BAME students. However, encouraging students with greater ethnic diversity into the profession will not remove the tensions experienced by the current BAME students unless it also supported by further education and understanding to address the existing tensions.

Finally, recognising that students from BAME backgrounds are not a homogenous group, we recommend that future research explores the specific experiences of particular ethnic or faith groups in physiotherapy education. Similarly, research exploring the experiences of qualified physiotherapists from these groups is also warranted.

## Data Availability

The datasets used and/or analysed during the current study are available from the corresponding author on reasonable request.

## Abbreviations

BAME: Black Asian and Minority Ethnic
BSc: Bachelor of Science
HEI: Higher Education Institution
HESA: Higher Education Statistics Agency
MSc: Master of Science
UK: United Kingdom
